# Weighted Gene Co-Expression Network Analysis Identifies Critical Genes in the Development of Heart Failure After Acute Myocardial Infarction

**DOI:** 10.3389/fgene.2019.01214

**Published:** 2019-11-26

**Authors:** Xiaowei Niu, Jingjing Zhang, Lanlan Zhang, Yangfan Hou, Shuangshuang Pu, Aiai Chu, Ming Bai, Zheng Zhang

**Affiliations:** ^1^Heart Center, The First Hospital of Lanzhou University, Lanzhou, China; ^2^Gansu Clinical Medical Research Center for Cardiovascular Diseases, The First Hospital of Lanzhou University, Lanzhou, China; ^3^Gansu Key Laboratory of Cardiovascular Diseases, The First Hospital of Lanzhou University, Lanzhou, China; ^4^The Quality Improvement Project for the Diagnosis and Treatment of Complicated Cardiovascular and Cerebrovascular Diseases (2018), The First Hospital of Lanzhou University, Lanzhou, China; ^5^Department of Internal Medicine, Baiyin Second People’s Hospital, Baiyin, China; ^6^Department of Digestive, The Second Affiliated Hospital of Xi’an Jiaotong University, Xi’an, China; ^7^The First School of Clinical Medicine, Lanzhou University, Lanzhou, China; ^8^Department of Cardiology, Gansu Provincial Hospital, Lanzhou, China

**Keywords:** acute myocardial infarction, heart failure, weighted gene co-expression network, key module, transcriptional factor, non-coding RNA, hub gene

## Abstract

**Background:** The development of heart failure (HF) remains a common complication following an acute myocardial infarction (AMI), and is associated with substantial adverse outcomes. However, the specific predictive biomarkers and candidate therapeutic targets for post-infarction HF have not been fully established. We sought to perform a weighted gene co-expression network analysis (WGCNA) to identify key modules, hub genes, and possible regulatory targets involved in the development of HF following AMI.

**Methods:** Genes exhibiting the most (top 50%) variation in expression levels across samples in a GSE59867 dataset were imported to the WGCNA. Gene Ontology and pathway enrichment analyses were performed on genes identified in the key module by Metascape. Gene regulatory networks were constructed using the microarray probe reannotation and bioinformatics database. Hub genes were screened out from the key module and validated using other datasets.

**Results:** A total of 10,265 most varied genes and six modules were identified between AMI patients who developed HF within 6 months of follow-up and those who did not. Specifically, the blue module was found to be the most significantly related to the development of post-infarction HF. Functional enrichment analysis revealed that the blue module was primarily associated with the inflammatory response, immune system, and apoptosis. Seven transcriptional factors, including SPI1, ZBTB7A, IRF8, PPARG, P65, KLF4, and Fos, were identified as potential regulators of the expression of genes identified in the blue module. Further, non-coding RNAs, including miR-142-3p and LINC00537, were identified as having close interactions with genes from the blue module. A total of six hub genes (*BCL3*, *HCK*, *PPIF*, *S100A9*, *SERPINA1*, and *TBC1D9B*) were identified and validated for their predictive value in identifying future HFs.

**Conclusions:** By using the WGCNA, we provide new insights into the underlying molecular mechanism and molecular markers correlated with HF development following an AMI, which may serve to improve risk stratification, therapeutic decisions, and prognosis prediction in AMI patients.

## Introduction

Acute myocardial infarction (AMI) is characterized by myocardial necrosis resulting from exposure to prolonged ischemia after occlusion of a coronary artery (Anderson and Morrow, 2017). With advances in interventional cardiology techniques, technologies, and pharmaceutical therapies, all-cause mortality has decreased during the acute phase of AMI over the past few decades (Anderson and Morrow, 2017). However, due to an increase in the number of patients surviving in hospitals after an AMI, and an increasingly aging global population, the incidence of heart failure (HF) is continuing to increase (Anderson and Morrow, 2017). The development of HF following an AMI has become a major public health concern because these patients exhibit the high rates of hospitalization and poor survival rates, comparable to that of cancer (Anderson and Morrow, 2017; Giustino et al., 2018).

The progression to HF after AMI is multifactorial and influenced by the extent of myocardial damage at the time of the index events as well as by the process of left ventricular remodeling (Maciejak et al., 2015; Giustino et al., 2018). A more comprehensive understanding of the mechanisms involved in development of HF following AMI will allow us to identify patients at risk and tailor individualized management regimens for each patient, ultimately reducing the socio-economic burden of HF. Several biomarkers are known to be associated with cardiac remodeling and the development of HF. For example, B-type natriuretic peptide and N-terminal pro-brain natriuretic peptide have been reported to exhibit strong prognostic values in patients with acute coronary syndromes in terms of the development of HF (Haeck et al., 2010). Inflammation biomarkers, leukocyte count, and neutrophil to lymphocyte ratio were independently predictive of HF and adverse events following an AMI (Seropian et al., 2016; Niu et al., 2018). However, robust early predictive methods for the development of HF following an AMI remain elusive.

Transcriptional profiling is an effective approach to provide biological insight and rapid, unbiased screening of nearly whole transcriptomes to reveal the most promising biomarkers for recognizing risk carriers. Moreover, some studies have identified the genes involved in AMI progression by using gene expression profiles (Qian et al., 2018). However, the previous studies were mostly concerned with differentially expressed genes (DEGs) and did not consider clusters of highly correlated genes, which may be responsible for specific clinical features of interests. Weighted gene co-expression network analysis (WGCNA) is a bioinformatics application for exploring the relationships between different gene sets (modules), or between gene sets and clinical features (Langfelder and Horvath, 2008). The WGCNA describes the correlation patterns between genes across microarray samples and provides straightforward biologically functional interpretations of gene network modules (Langfelder and Horvath, 2008). Currently, the WGCNA has been successfully used to construct gene co-expression networks in various diseases, most notably in different cancers, and identify centrally connected hub genes as promising biomarkers or therapeutic targets (Chen et al., 2016; Li et al., 2019b; Wang et al., 2019). Additionally, the use of WGCNA can provide novel insights into which functional regulators may be driving transcriptional signatures in the development of AMI, such as transcription factors (TFs) (Chen et al., 2016), microRNAs (miRNAs) (Li et al., 2019b), and long non-coding RNAs (lncRNAs) (Wang et al., 2019). To date, there are few studies identifying the biomarkers that are functionally implicated in ventricular remodeling and with the ability to predict HF development following an AMI using the WGCNA algorithm.

In the present study, the WGCNA was constructed based on data from the discovery dataset GSE59867 obtained from the Gene Expression Omnibus (GEO) database. Key gene modules associated with the development of HF after AMI were identified, and the biological functions and pathways of genes in the key modules were analyzed. Hub genes in the key module were detected using other datasets from the GEO database, namely the validation dataset GSE42955. The diagnostic performance of the identified hub genes for HF development after AMI was evaluated by receiver operating characteristic (ROC) curve analysis of dataset GSE1869. Information on gene expression patterns associated with AMI during a follow-up of 6 months was also revealed. Furthermore, since TFs, miRNAs, and lncRNAs play important regulatory roles in human diseases, detailed analyses of these regulators will allow us to better understand the molecular mechanisms underlying post-infarction HF. We constructed TF-gene regulation networks, miRNA-target regulatory networks, and lncRNA–mRNA co-expression patterns for the key module. Based on the bioinformatic analyses, our study is expected to provide novel insights into the pathogenesis and progression of AMI, and identify distinct biomarkers that correlate with HF development.

## Materials and Methods

### Data Sources

**Figure 1:**
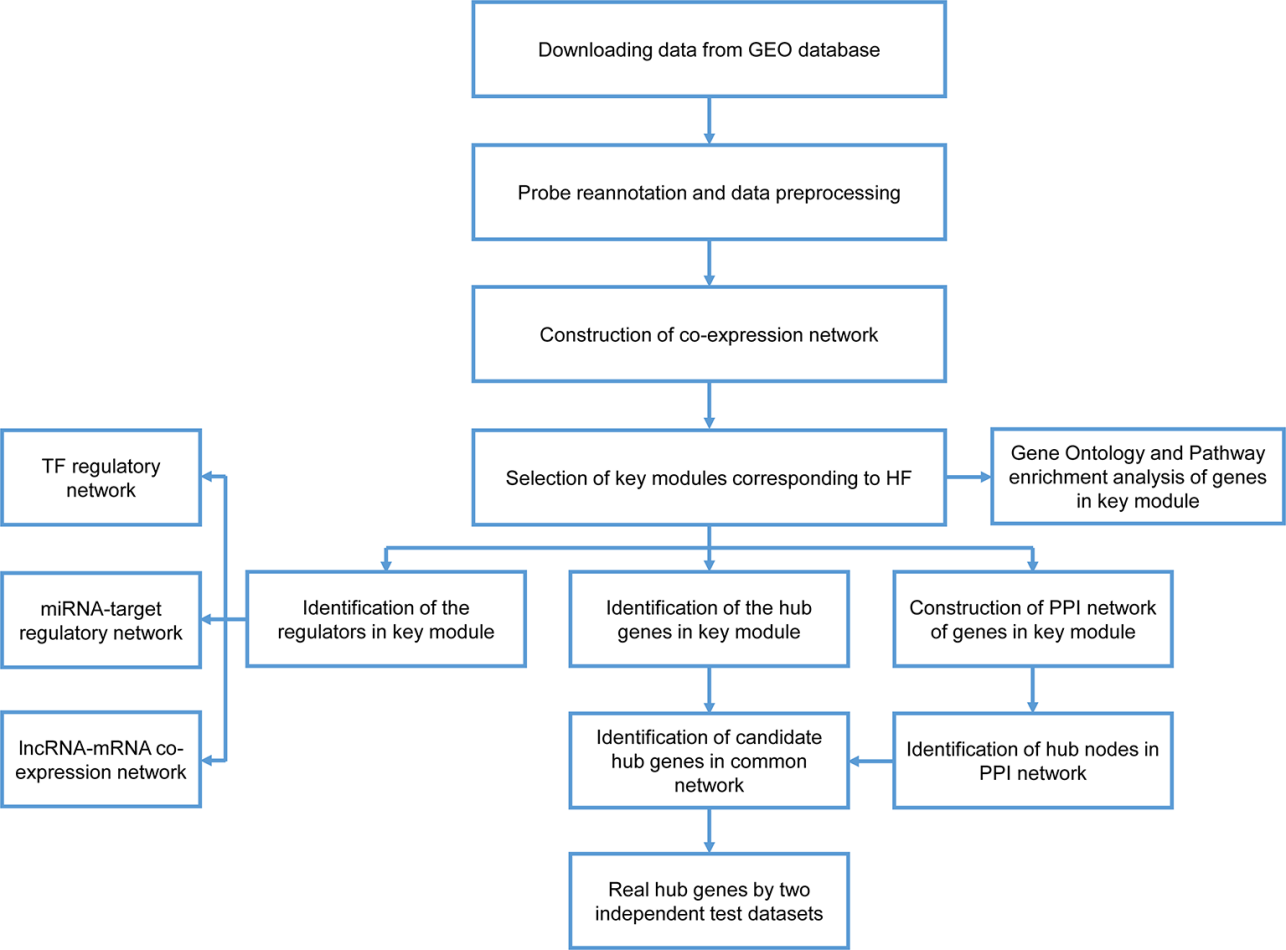
Flow chart of data preparation, processing, analysis, and validation.

A workflow for this study was presented in Figure 1. The datasets of GSE59867, GSE1869, and GSE42955 were obtained from the GEO database (http://www.ncbi.nlm.nih.gov/geo/). In the GSE59867 dataset (Maciejak et al., 2015), details on the development of HF during the 6-month follow-up were recorded for 65 samples obtained from 17 patients with AMI. The transcriptional profiling of peripheral blood mononuclear cells (PBMCs) in these 17 patients, which were performed at admission, discharge (4–6 days), 1 month, and 6 months after AMI, were selected for further analysis. There were no significant differences observed in the baseline demographic and clinical characteristics between HF (n = 9) and non-HF (n = 8) patients. This data were sequenced using the GPL6244 platform [Affymetrix Human Gene 1.0 ST Array, transcript (gene) version]. The GSE1869 dataset included 10 patients with HF post-AMI and 6 non-HF patients, and was performed using the platform GPL96 (Affymetrix U133A microarray) (Kittleson et al., 2005). The GSE42955 dataset included 12 patients with HF post-AMI and 5 non-HF patients, and was performed using the platform GPL6244 (Molina-Navarro et al., 2013).

### Probe Reannotation

The lncRNA expression data were obtained by reannotating the probes strategy according to previous studies (Zhang et al., 2012; Wang et al., 2017). Briefly, probe sets were mapped to RefSeq transcript IDs and/or Ensembl gene IDs based on the latest version of the NetAffx Annotation File (HuGene-1_0-st-v1 Probeset Annotations, CSV Format, Release 36). To make the screened lncRNAs be more reliable (Zhang et al., 2012; Wang et al., 2017), only probe sets that were labeled “NR” in the Refseq database and annotated with the non-coding RNA titles in the Ensembl database were retained, which resulted in 514 annotated lncRNA genes. If more than one probe corresponded to one gene, the expression value of that gene was computed by determining the median expression value of all its corresponding probes.

### Data Preprocessing

The data from the gene expression profiling analysis were preprocessed using Robust Multichip Average algorithm in the affy package within Bioconductor (http://www.bioconductor.org) in the R 3.3.1 software (R Foundation for Statistical Computing, Vienna, Austria). After correcting for background, and performing quantile normalization and log2-transformation, the data set containing 20,530 genes was further processed, and the 10,265 genes exhibiting the top 50% in high expression variance (minimize the loss of statistical information) were selected for the WGCNA (Li et al., 2017a).

### Construction of WGCNA

The R package, WGCNA, was used to perform the weighted correlation network analysis (Langfelder and Horvath, 2008). Firstly, the gene co-expression similarity between genes m and n was defined as S_mn_ = |cor(m, n)|. A power function was then applied to correlate adjacency of genes: a_mn_ = power (S_mn_, β) = |S_mn_|^β^. Scale independence and mean connectivity were then tested using a gradient method (the power value ranging from 1 to 20). When the degree of independence was determined to be above 0.80 (Langfelder and Horvath, 2008), an appropriate power value was screened out to obtain a scale-free network. Finally, the adjacency matrix was transformed into a topological overlap matrix, and modules were detected by hierarchical average linkage clustering analysis for the gene dendrogram. Additionally, we extracted the corresponding gene information for each module for further analysis.

### Selection of Key Modules Corresponding to Clinical Traits

After the modules were identified, the module eigengene (ME) was summarized by the first principal component of the module expression levels. Module–trait relationships were estimated using the correlation between MEs and clinical traits, which allowed efficient identification of the relevant modules. To evaluate the correlation strength, we calculated the module significance (MS), which is defined as the average absolute gene significance (GS) of all the genes involved in the module. The GS is measured as the log10 transformation of the P value (lgP) in the linear regression between gene expression and clinical information. In the WGCNA, modules with the highest MS score among all modules are usually defined as the key module and selected for further analysis (Langfelder and Horvath, 2008; Wright et al., 2015).

### Functional Enrichment Analysis of the Key Module

To understand the biological meaning of the key module, the gene information was loaded into Metascape (http://metascape.org) for Gene Ontology (GO) enrichment analysis (Zhou et al., 2019). Pathway enrichment analysis was carried out with the following ontology sources: Kyoto Encyclopedia of Genes and Genomes (KEGG) Pathway and Molecular Signatures Database (MSigDB) Hallmark Gene Sets (Zhou et al., 2019). Terms with a P value <0.05, a minimum count of 3, and an enrichment factor >1.5 were collected and grouped into clusters based on their membership similarities (Kappa scores >0.3) (Zhou et al., 2019). The most statistically significant term within a cluster was chosen to represent the cluster. If more than 20 terms for GO or pathway annotations were identified, the top 20 terms were chosen for visualization.

### Identification of TFs in the Key Module

Enrichr (http://amp.pharm.mssm.edu/Enrichr/) is a comprehensive web-based tool that contains 180,184 annotated gene sets from 102 gene set libraries (Kuleshov et al., 2016). The gene information for the key module was imported into the Enrichr to obtain the interaction between transcription factors (TFs) and their target genes. To reduce the chance of identifying false-positives, we extracted TFs with consensus target genes existing in ChEA, ENCODE gene-set library, and position weight matrices from TRANSFAC and JASPAR. We then used the Cytoscape 3.4.0 software (Cytoscape Consortium, San Diego, CA, USA) to visualize the TF-target gene regulatory networks.

### Identification of miRNAs in the Key Module

Two different miRNA target-predicting algorithms within the Enrichr tool, including TargetScan (Agarwal et al., 2015) and miRTarBase (Chou et al., 2018), were employed to screen potential miRNAs that regulate the genes in key modules. The TargetScan predicts biological targets of miRNAs by searching for the presence of mRNA sites that match the seed region of each miRNA (Agarwal et al., 2015). The miRTarBase is a curated database which has accumulated more than 50,000 miRNA–target interactions which are validated experimentally by reporter assay, western blot, microarray, and next-generation sequencing experiments (Chou et al., 2018). The common identified miRNAs were then used to construct miRNA–mRNA pairs. The regulatory association was displayed by the Cytoscape software.

### Identification of lncRNAs in the Key Module

Hub genes that are highly interconnected with nodes in a module have been shown to be functionally significant. Module membership (MM) represents how close a gene’s expression conforms to the characteristics of the module. The MM was calculated as correlation between individual gene expression values and ME. In this study, lncRNAs with highly intramodular connectivity (MM ≥ median) were retained to form connections of lncRNAs and mRNAs (Lunnon et al., 2012). The Pearson correlation coefficients (r) for the RNAs in the key module were calculated again to construct the lncRNA–mRNA co-expression network. Finally, the significant lncRNA–mRNA pairs (|r| ≥ median and P < 0.05) were visualized using the Cytoscape software.

### Identification of Candidate Hub Genes in the Key Module

Candidate hub genes were screened out using module connectivity, measured by MM ≥ median and clinical trait relationships, measured by GS ≥ median (Lunnon et al., 2012). To identify experimentally validated interactions in the key module, we uploaded all genes identified in the key module to the Search Tool for the Retrieval of Interacting Genes (STRING) database. Only protein–protein interactions (PPI) based on experiments with a combined score 0.4 were selected as significant. The subnetworks of PPI were then constructed with the plug-in MCODE in the Cytoscape (degree cut-off ≥2, node score cut-off ≥0.2, K-core ≥2, and max depth = 100). Genes identified in the MCODE subnetworks and exhibiting high MM and high GS in the co-expression network were chosen as the candidates for further analysis and validation.

### Identification of Real Hub Genes

The independent datasets GSE1869 and GSE42955 from the GEO database were extracted, and data were preprocessed by correcting for background and performing quantile normalization and log2-transformation. Another WGCNA using dataset GSE42955 were performed to validate the candidate hub genes. In the validation set GSE42955, a total of 11,653 genes exhibiting the top 50% in high expression variance were used for the WGCNA. The module with the maximal MS score among all modules was selected as the most relevant module in GSE42955. Genes with MM ≥ median and GS ≥ median in the relevant module were mapped to the candidate hub genes.

In GSE1869, the area under the curve (AUC) of the ROC was calculated to evaluate the diagnostic accuracy of the candidate hub genes mapped to genes in GSE42955. The AUC is the value of the Wilcoxon–Mann–Whitney statistic, and 95% confidence interval (CI) for AUC was computed using the standard normal distribution (Gengsheng and Hotilovac, 2008). Genes with the AUC ≥0.80 (P < 0.05) in ROC curve analysis represented clinically relevant genes and were defined as the real hub genes.

## Results

### Construction of Weighted Gene Co-Expression Network

After preprocessing of the GSE59867 dataset, the microarray quality was evaluated by sample clustering according to the distance between different samples observed in Pearson’s correlation matrices. No outliers were detected in the clusters, and therefore 65 samples were used to construct a hierarchical clustering tree (dendrogram) (**Supplementary Figure S1A**). Next, a power of β = 12 was selected as the soft-threshold to ensure a scale-free network (**Supplementary Figures S1B, C**). As a result, 10,265 genes were grouped into a total of six modules using the average linkage hierarchical clustering algorithm.

As shown in **Supplementary Figure S2**, 2,933 genes to the blue module, 889 genes to the brown module, 170 genes to the green module, 4,753 genes to the turquoise module, and 332 genes to the yellow module. The genes that were not grouped into a module were included in the grey module, which was removed during the subsequent analysis.

### Construction of Module–Trait Relationships and Detection of Key Modules

**Figure 2:**
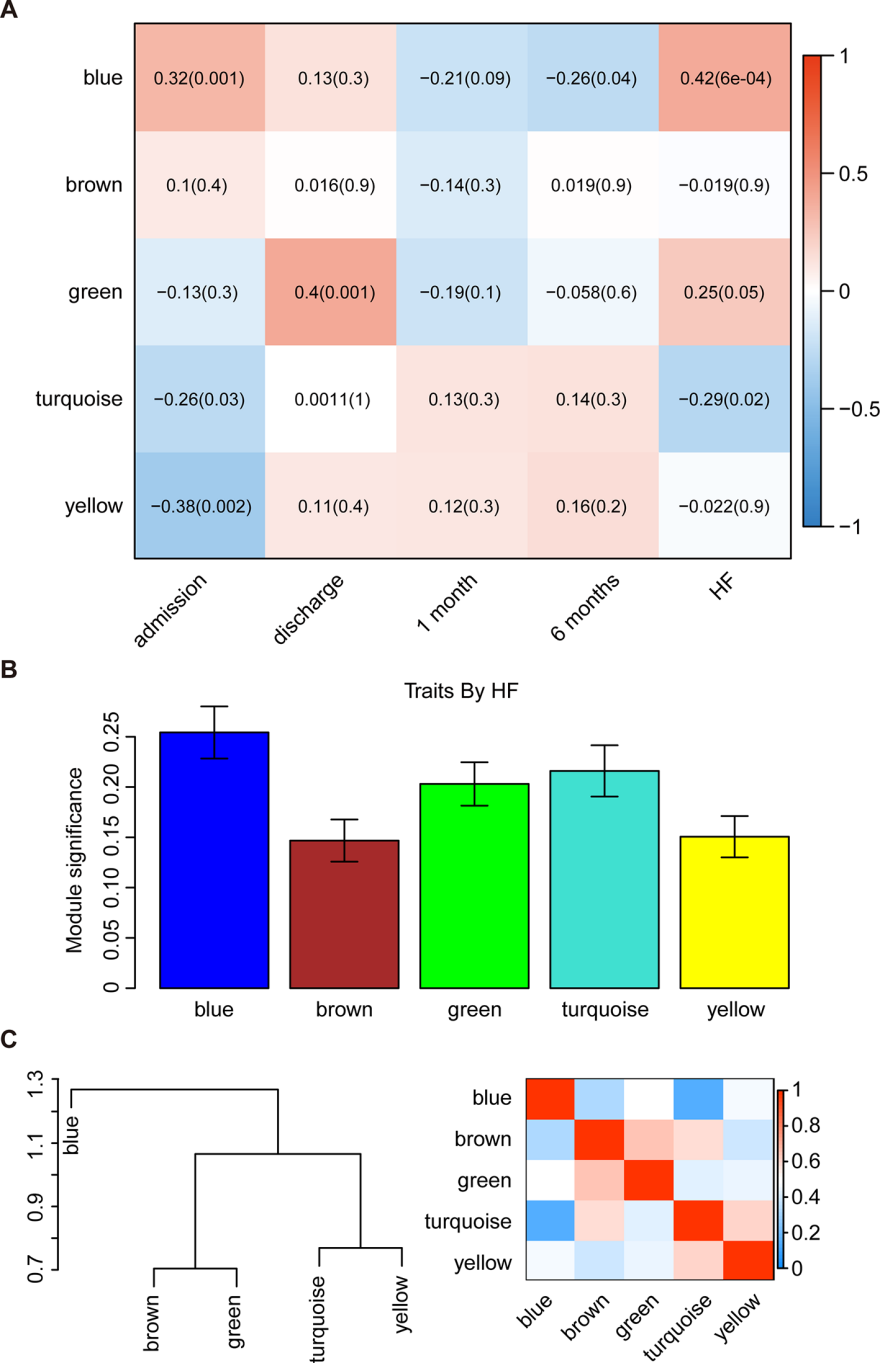
Identification of the key modules associated with the development of HF after AMI. **(A)** Heatmap depicting the correlation between module eigengenes and clinical traits of AMI. **(B)** Distribution of module significance and errors in the modules associated with the development of HF after AMI. **(C)** Hierarchical clustering dendrogram showing the module eigengenes and a heatmap of the adjacencies in the eigengene network (labeled according to by their module color names).

The WGCNA was then used to correlate each module with all available clinical information (time points following AMI and HF progression) in the GSE59867 dataset by calculating the MS for each module–trait correlation (**Figures 2A, B**). After screening for strong correlations between all modules and HF progression, we found that the blue module had the highest MS value among all the selected modules. The ME in the blue module also exhibited a higher correlation with HF progression than other modules (R^2^ = 0.42 and P < 0.001). Additionally, we found that genes in the blue module were significantly affected on the first day of AMI (day of admission).

Interaction-based relationships for the five modules were illustrated in **Figure 2C**. The results revealed that the five modules were primarily divided into two clusters according to their ME correlation. Similar results were demonstrated using a heatmap, which showed the adjacencies in the eigengene network, suggesting a high level of independence among the modules. Therefore, the blue module was considered to be the key module, and was, therefore, chosen for further analysis.

### Functional Enrichment Analysis of Genes in the Blue Module

**Figure 3:**
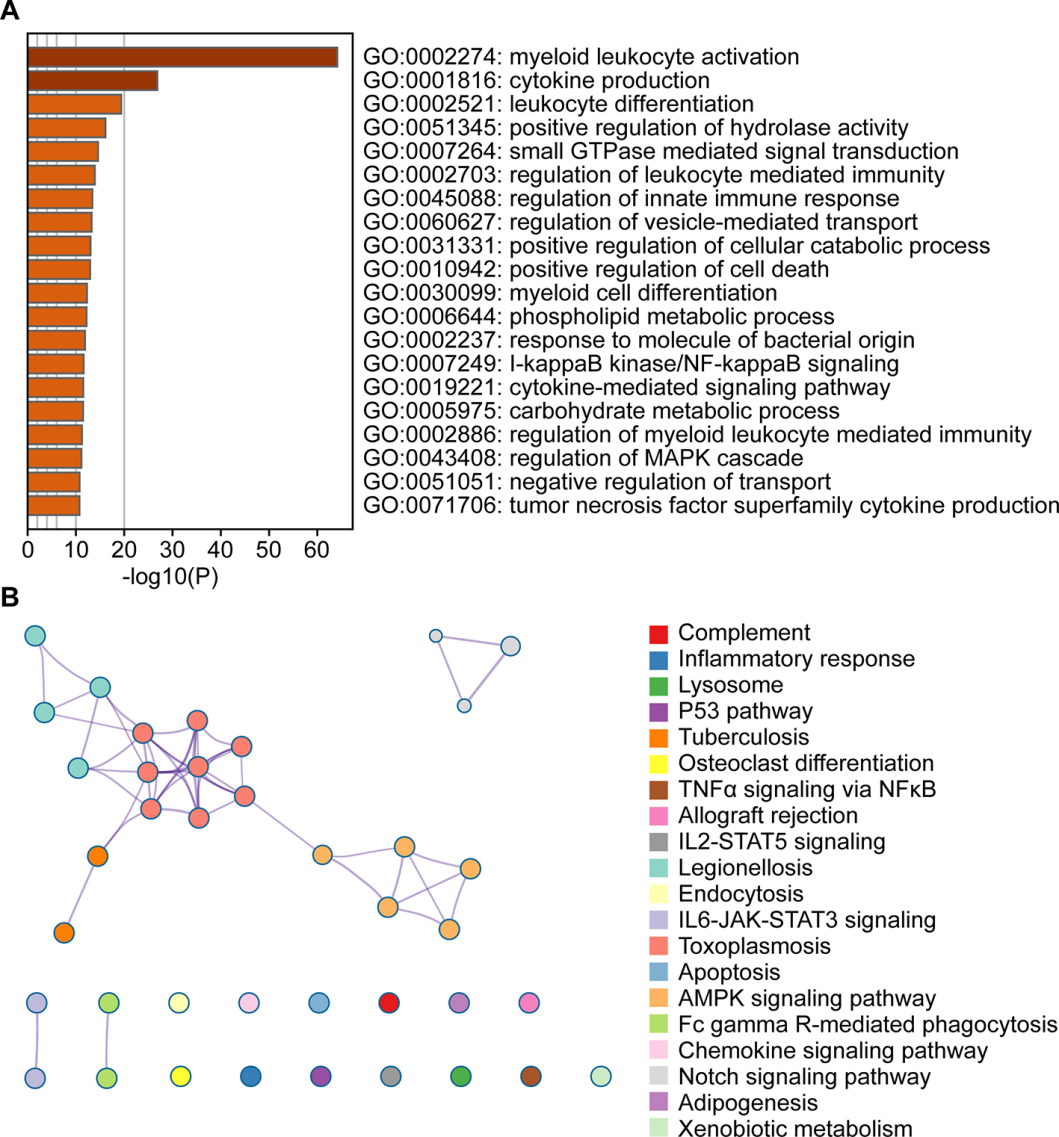
Functional enrichment analysis of the blue module genes. **(A)** Gene Ontology analysis of genes in the blue module. **(B)** KEGG pathway enrichment analysis of genes in the blue module.

To evaluate the affected functions for the genes clustered in the blue module, we performed GO and pathway analyses. We determined that these enriched pathways were closely connected with each other. The enriched results for the significant functions and pathways were presented in **Figures 3A, B**.

### TF Regulatory Network

**Figure 4:**
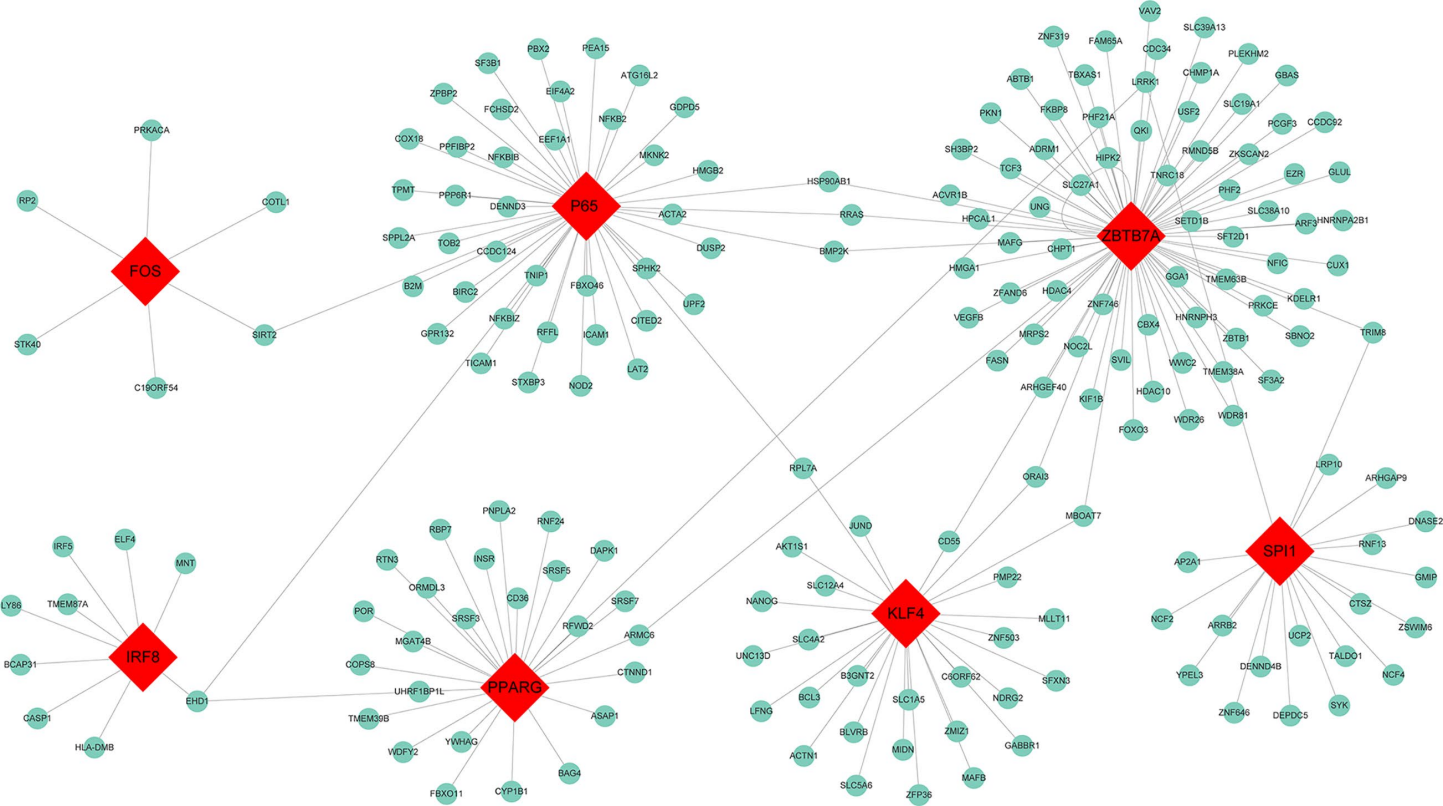
Transcription factor regulatory network for the genes in the blue module. Red diamonds represent the transcription factors, and green nodes represent the genes.

To determine whether TFs may be responsible for the observed altered gene expression in the blue module, we inspected four data sources available in Enrichr, namely ENCODE, ChEA, TRANSFAC, and JASPAR. A total of 31 TFs were identified and seven among them were found to be present in the blue module, including Spi-1 proto-oncogene (SPI1) which had 20 target genes identified, zinc finger and BTB domain containing 7A (ZBTB7A) with 76 target genes, interferon regulatory factor 8 (IRF8) with 9 target genes, peroxisome proliferator activated receptor gamma (PPARG) with 27 target genes, P65 with 44 target genes, Kruppel like factor 4 (KLF4) with 29 target genes, and Fos proto-oncogene AP-1 transcription factor subunit (Fos) with 6 target genes. The TF-target gene regulatory network was displayed in **Figure 4**.

### miRNA-Target Regulatory Network

**Figure 5:**
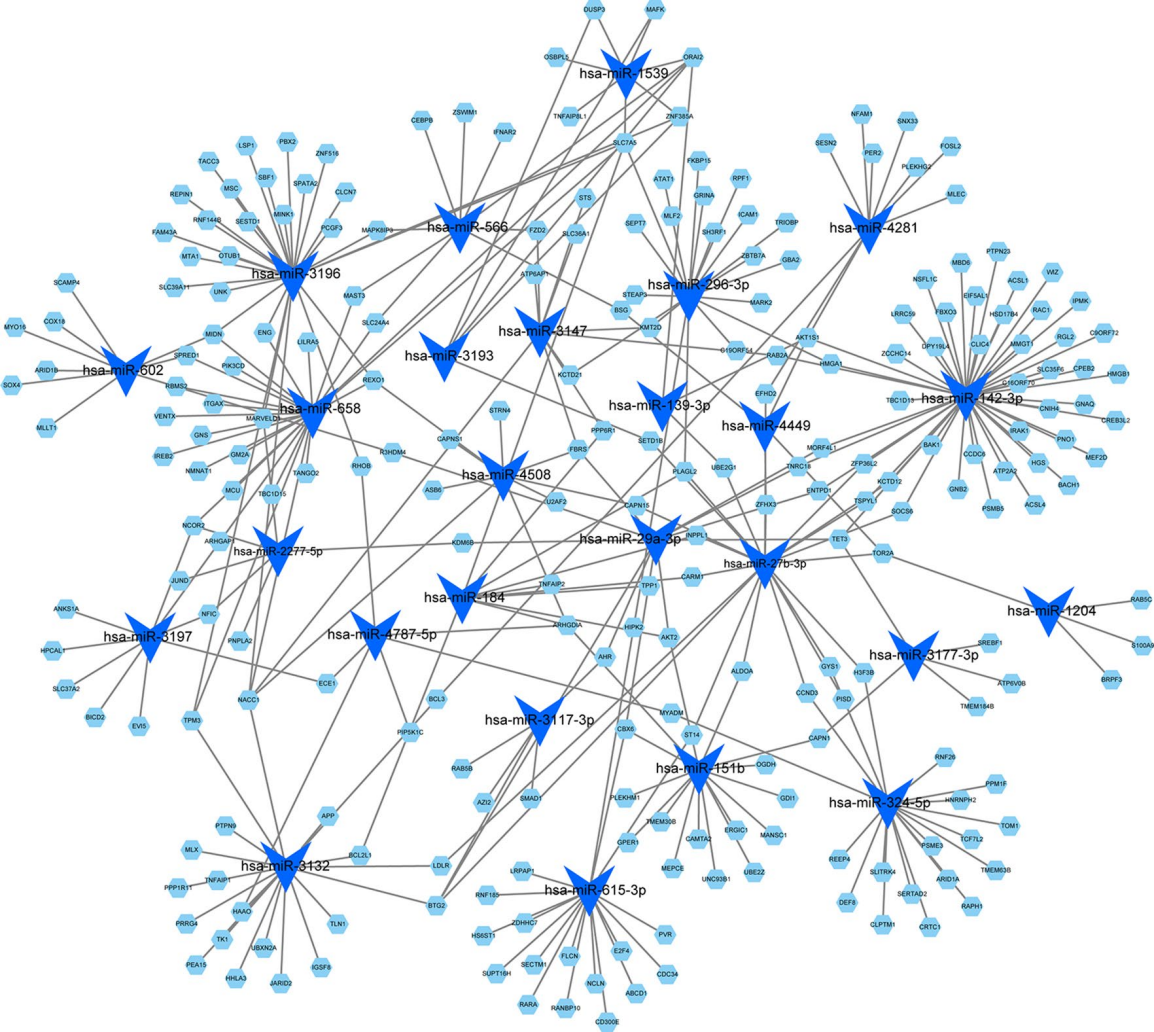
MicroRNA-target regulatory network for the blue module. Blue triangles represent the microRNAs, and green nodes represent the genes. The node size indicates the degree in the network.

By using the TargetScan and miRTarBase databases, we were able to identify the 26 most common miRNAs responsible for regulating the target genes in the blue module (**Figure 5**). The greatest number of genes were regulated by miR-142-3p (degree = 47). Thus, these results indicated that miR-142-3p may serve a role in HF progression.

### lncRNA–mRNA Co-Expression Network

**Figure 6:**
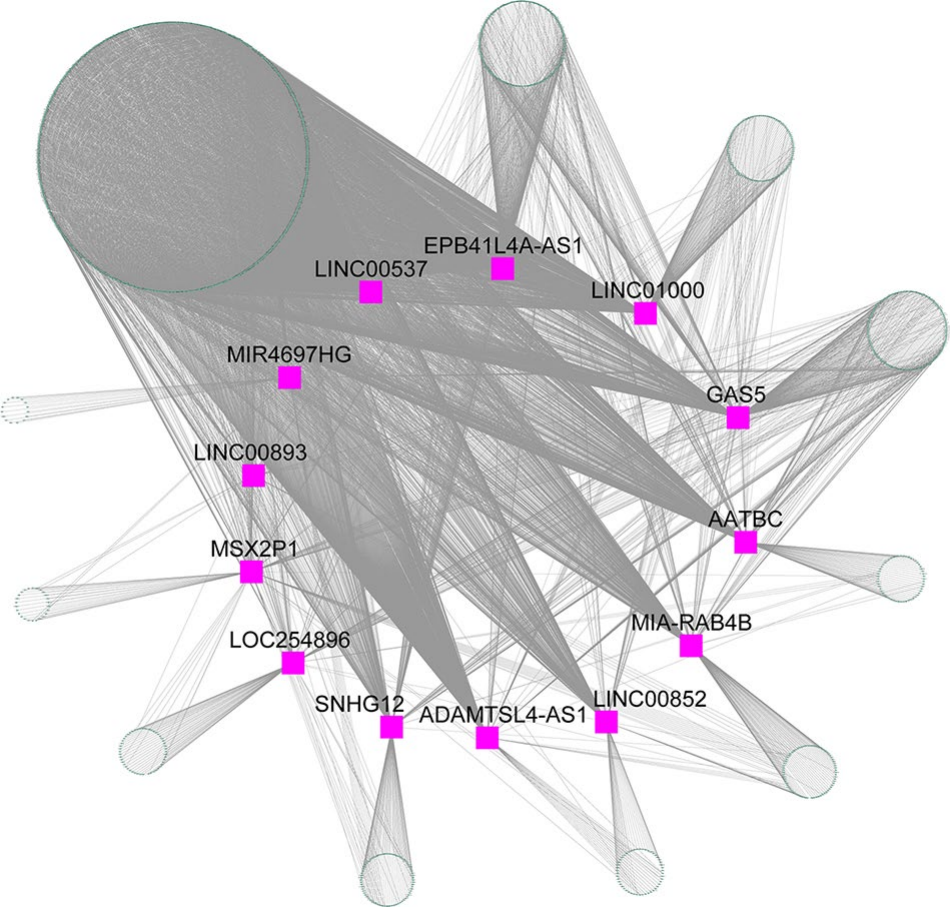
lncRNA-mRNA co-expression network for the blue module. Purple squares represent the lncRNA, and green nodes represent the genes.

The blue module contained 2,892 mRNAs and 41 lncRNAs. We extracted 13 lncRNAs with high MM (> 0.60) to calculate the |r| for each interaction pair, which included apoptosis associated transcript in bladder cancer (AATBC), ADAMTSL4 antisense RNA 1 (ADAMTSL4-AS1), EPB41L4A antisense RNA 1 (EPB41L4A-AS1), growth arrest specific 5 (GAS5), long intergenic non-protein coding RNA 00537 (LINC00537), long intergenic non-protein coding RNA 00852 (LINC00852), long intergenic non-protein coding RNA 00893 (LINC00893), long intergenic non-protein coding RNA 1000 (LINC01000), LOC254896, MIA-RAB4B readthrough (NMD candidate) (MIA-RAB4B), MIR4697 host gene (MIR4697HG), msh homeobox 2 pseudogene 1 (MSX2P1), and small nucleolar RNA host gene 12 (SNHG12). The gene pairs with |r| higher than 0.60 (P < 0.05) were retained to construct the lncRNA-mRNA co-expression network. As shown in **Figure 6**, the 13 lncRNAs were found to be functional associated with more than one gene. LINC00537 was found to be strongly connected (|r| range from 0.60 to 0.82) with the highest degree of connectivity (760) in the co-expression network.

### Selection of Candidate Hub Genes

**Figure 7:**
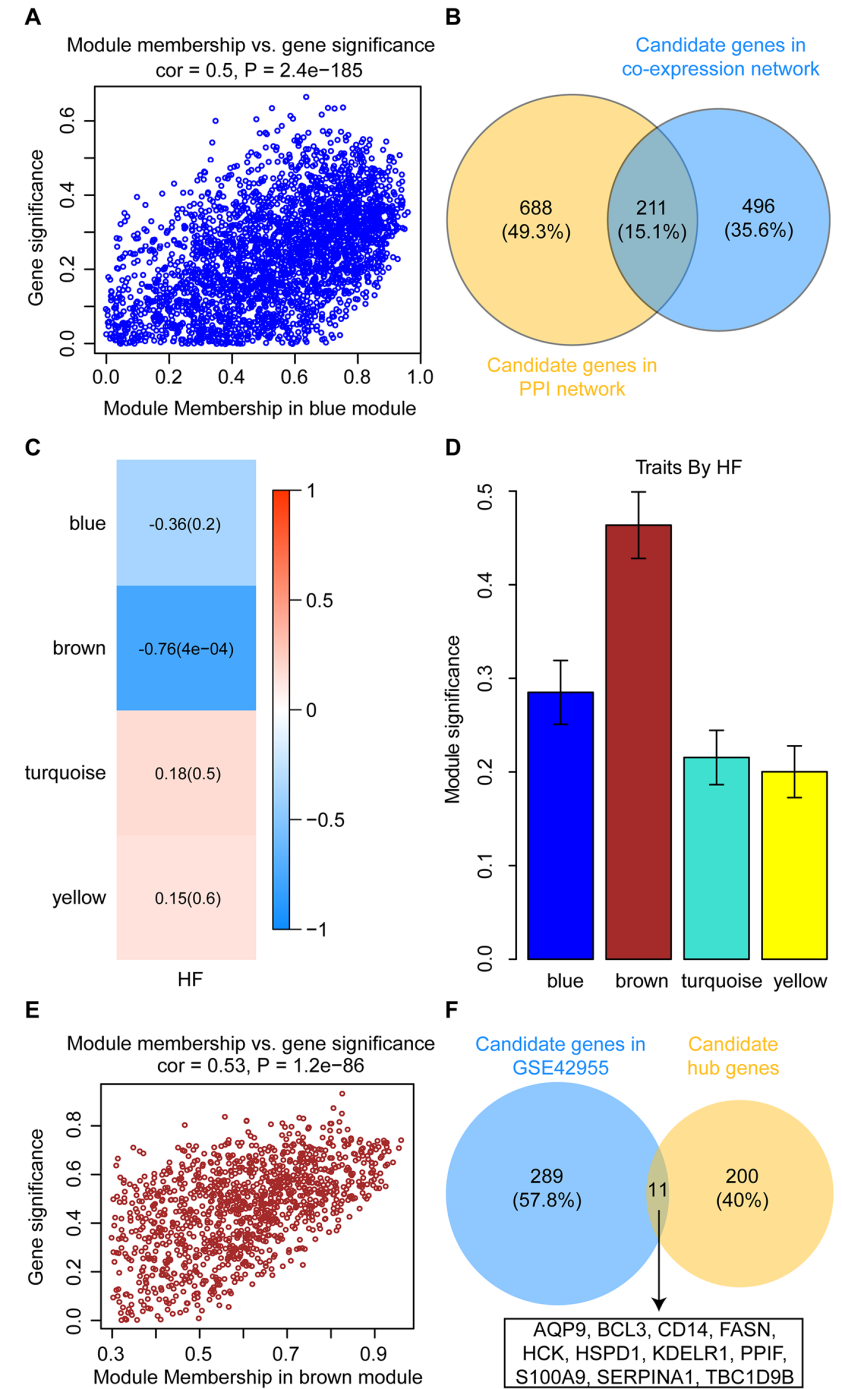
Identification and validation of candidate hub genes. **(A)** Scatterplot of gene significance versus module membership for the development of HF after AMI in the blue module. **(B)** Common genes between the co-expression network (GSE59867) and the MCODE sub-module of PPI network. **(C)** Heatmap depicting the correlation between module eigengenes and HF progression in the GSE42955 dataset. **(D)** Distribution of module significance and errors in the modules associated with the development of HF after AMI in the GSE42955 dataset. **(E)** Scatterplot of gene significance versus module membership for the development of HF after AMI in the brown module (GSE42955). **(F)** Common hub genes between the co-expression network (GSE42955) and the candidate hub genes.

To identify intramodular hub genes, we plotted the MM against the GS for traits that correlated with MEs. Under the condition of higher-than-median MM [≥0.60 (range 0.10–0.96)] and GS (≥ 0.24), 707 genes in the blue module were retained for further analysis (**Figure 7A**). The PPI network were constructed in the STRING database, which included a total of 2,520 nodes and 18,133 interaction pairs. Meanwhile, MCODE analysis was performed on the PPI network and an additional 899 genes, with high connective degrees, were filtered out (**Figure 7B**). A total of 211 candidate genes, identified both in the MCODE subnetworks and co-expression network, were included as potential intramodular hub genes (**Figure 7B**).

### Identification of Real Hub Genes

In the GSE42955 dataset, 16 samples were used to construct a hierarchical clustering tree (dendrogram) (**Supplementary Figure S3A**). Next, a power of β = 14 was selected as the soft-threshold to ensure a scale-free network (**Supplementary Figures S3B, C**). The 11,653 genes were grouped into a total of five modules using the average linkage hierarchical clustering algorithm. As shown in **Supplementary Figure S4**, 2,355 genes to the blue module, 1,183 genes to the brown module, 5,817 genes to the turquoise module, and 1,165 genes to the yellow module. After screening for strong correlations between all modules and HF progression, we found that the brown module had the highest MS and ME values among all the selected modules (**Figures 7C, D**). Under the condition of higher-than-median MM [≥0.60 (range 0.30–0.96)] and GS (≥ 0.49), 300 genes in the brown module were retained for further analysis (**Figure 7E**). All candidate hub genes were mapped to the 300 genes in the GSE42955 datasets, and 11 genes of these candidate hub genes were validated (**Figure 7F**).

**Figure 8:**
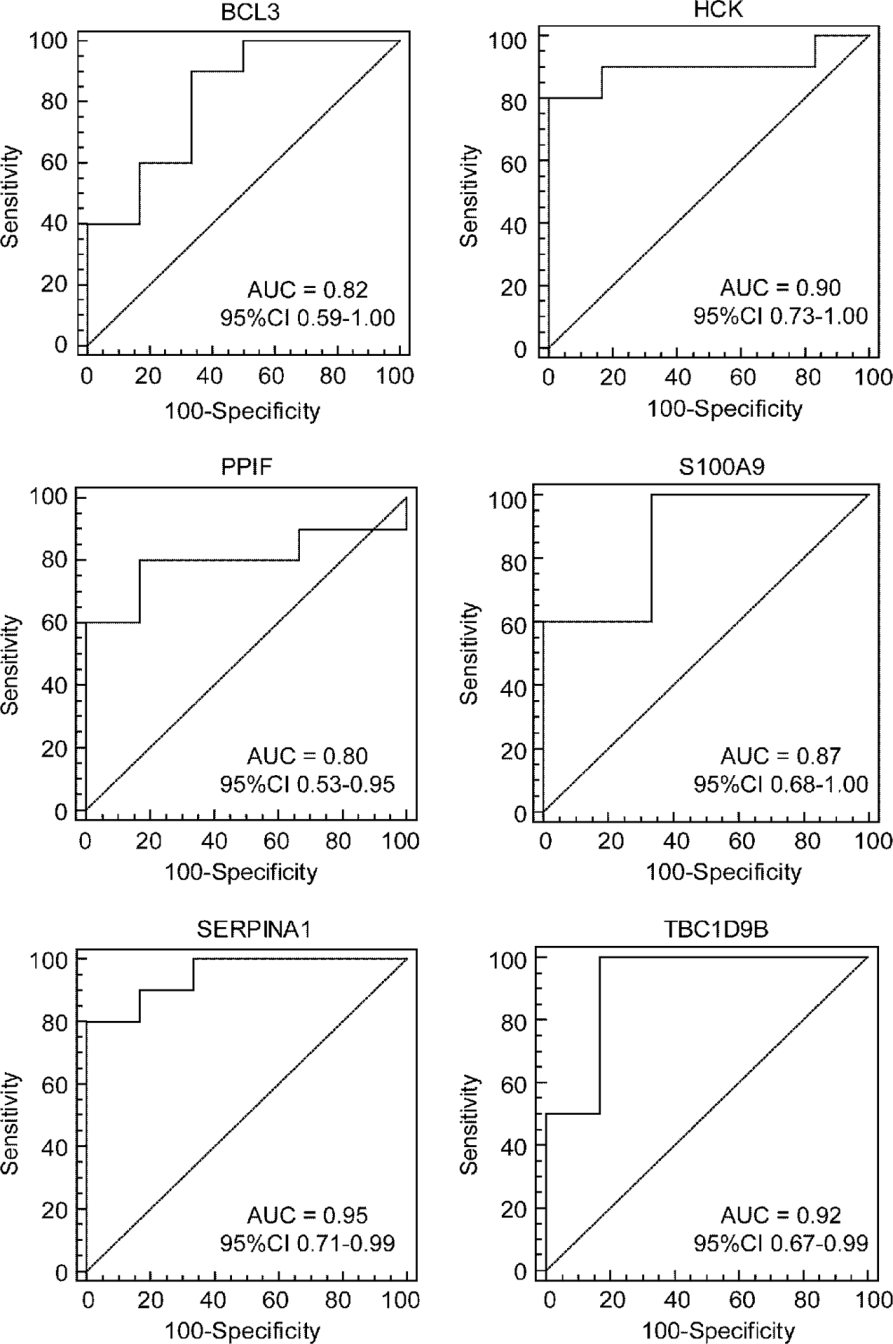
Identification of real hub genes. ROC curve analysis of the candidate hub genes in the GSE1869 dataset.

To further test the value of the candidate hub genes as prognostic biomarkers of HF, ROC curves were performed and the AUC (95% CIs) were calculated (**Figure 8**). The results indicated that six genes exhibited a high predictive accuracy for the development of HF after AMI, including B-cell leukemia/lymphoma 3 (*BCL3*), hematopoietic cell kinase (*HCK*), peptidylprolyl isomerase F (*PPIF*), S100 calcium binding protein A9 (*S100A9*), serpin family A member 1 (*SERPINA1*), and TBC1 domain family member 9B (*TBC1D9B*). Hence, these six genes were regarded as the real hub genes in the WGCNA.

## Discussion

In the present study, we applied the WGCNA to identify biologically relevant transcripts significantly altered upon experiencing an AMI and throughout the subsequent clinical follow-up. We further identified the key modules and regulators of gene expression that were unique to patients who developed HF after AMI, and evaluated the usefulness of hub genes as prognostic biomarkers associated with post-infarction HF.

Compared with the previous studies, our investigation provides new insights into the pathogenesis of HF following AMI. Chen et al. analyzed DEGs between the AMI group and the healthy control group using the dataset GSE48060 (Chen et al., 2016). They revealed that TBX21 and PRF1 may be potential candidates for diagnostic biomarker in AMI. Similarly, Zhang et al. used the dataset GSE48060 to screen potential biomarkers for AMI development (Zhang et al., 2017b). Huan et al. analyzed coronary heart disease-related genes by constructing co-expression networks (Huan et al., 2013). In contrast, our study identified the key genes between AMI patients who developed HF within 6 months of follow-up and those who did not. Pang et al. constructed a comprehensive transcriptome profile using RNA-seq and miRNA-seq data of 16 patients with HF and 8 non-failing individuals (Pang et al., 2016). Their results provided deep insights into the critical roles of lncRNAs in the pathology of HF. However, the genes identified by Pang et al. came from patients with HF including ischemic and non-ischemic causes, which have been shown to be heterogeneous in terms of clinical presentation and prognosis (Zimmer et al., 2019). Our study identified the key genes in HF post-AMI, and determined the role the key genes as early prognostic biomarkers.

Transcriptional profiling has recently become a promising tool to investigate the specific molecular mechanisms underlying cardiovascular diseases, and to identify accurate biomarkers for disease progression (Vausort et al., 2014). Transcriptomic analysis of cardiac tissues would more accurately describe the etiology and pathophysiology of HF. However, biopsies may not always be a feasible option for patients with AMI. Peripheral blood cells are therefore an attractive alternative to cardiac biopsies. Numerous studies have demonstrated that PBMCs play important roles in the systemic and local inflammation associated with pathological remodeling after AMI (Epelman et al., 2015; Zimmer et al., 2019). Moreover, gene expression profiles of PBMCs and cardiomyocytes share common features in response to aldosterone treatment in hypertensive rats (Gerling et al., 2013), suggesting that transcriptional signatures of PBMCs may serve as early sentinels predictive of pathological remodeling. In addition to being non-invasive and convenient, the use of blood as a surrogate tissue has proven valuable in effectively revealing gene signatures for the development of diagnostic and prognostic biomarkers in coronary artery disease (Wingrove et al., 2008) and chronic HF (Cappuzzello et al., 2009).

AMI is the primary contributor of HF and loss of life among cardiovascular diseases (Anderson and Morrow, 2017). It is of clinical importance to identify factors associated with the molecular predisposition for developing HF to identify AMI patients requiring special care and treatment (Anderson and Morrow, 2017). In the WGCNA, we identified five unique gene modules based on gene expression profiling in PBMCs. The blue module was found to be most significantly related to the HF status in patients with AMI. The gene changes within the blue module were strongly induced on the first day of AMI, upon admission to the health care facility. These results suggested that the extent of myocardial damage during the acute phase is closely associated with the stable heart function progressing into HF (Epelman et al., 2015; Zimmer et al., 2019). The identified transcripts present during the first day of AMI can serve as a tool contributing to early diagnosis of the development of HF after AMI.

To comprehensively investigate the functions and pathways affected by genes in the blue module, a functional enrichment analysis was performed. We found that the genes in the blue module were significantly enriched in immunity and inflammation-related biological processes. These results were in accordance with previous reports which showed that the DEGs identified between HF and non-HF groups were highly associated with inflammatory-immune responses (Qian et al., 2018). Furthermore, our pathway analysis revealed that most annotated transcripts were associated with pathways related to inflammatory responses, the immune system, signal transduction, and apoptosis. Consistent with previous studies (Zhang et al., 2013), we also demonstrated that IL-6-JAK-STAT3 signaling played an important role in the pathogenesis of AMI. Multiple studies have suggested that high levels of pro-inflammatory cytokines like TNFα and IL-6 can contribute toward cardiac dysfunction and failure (Schumacher and Naga Prasad, 2018). The excessive apoptosis of myocardial cells has been demonstrated to induce the development of cardiac dysfunction after AMI (Zimmer et al., 2019). Therefore, these altered genes in the blue module are likely linked with the more severe initial injury to the myocardium which culminates in later stages of HF.

TFs are regulators of gene expression that are closely associated with the development and progression of post-infarction HF. In the current study, we assessed the effect of TFs on the expression of genes within the blue module, and identified seven TFs including SPI1, ZBTB7A, IRF8, PPARG, P65, KLF4, and Fos as significantly associated with these genes. Similarly, Qiao’s study also reported that SPI1, ZBTB7A, and IRF8 play crucial roles in the development of dilated cardiomyopathy and ischemic cardiomyopathy by regulating inflammation- and apoptosis-related genes (Qiao et al., 2017). The *SPI1* gene encodes an ETS-domain TF that is involved in the differentiation or activation of macrophages and dendritic cells (Yashiro et al., 2019). Additionally, a recent study (Fischer et al., 2019) demonstrated that SPI1 shapes the neutrophil epigenome by positively, and negatively regulating distinct immune-associated gene sets, thereby protecting the host from undergoing uncontrolled activation of immune responses. Further, an RNA sequencing study examining cardiac hypertrophy demonstrated that SPI1 was significantly up-regulated in the pathogenesis of adverse myocardial remodeling (Song et al., 2012). ZBTB7A is a POZ-domain-containing protein that directly binds to many genomic regulatory sites and regulates transcription both by controlling chromatin structure and through recruiting TFs to genomic promoters and enhancers (Ramos Pittol et al., 2018). ZBTB7A has been reported to activate the transcription of nuclear factor kappa B (NF-κB)-induced genes by enhancing DNA accessibility in chromatin (Ramos Pittol et al., 2018). IRF8 is a TF from the interferon regulatory factor (IRF) family that is induced by interferon in a variety of cell types, such as macrophages and T cells (Jiang et al., 2014). IRF8 acts as a transcriptional activator or repressor through the formation of different DNA-binding heterocomplexes with multiple partners, including members of the IRF family (IRF1, IRF2, and IRF4) and non-IRF transcription factors (SPI1 and ZBTB17) (Jiang et al., 2014). Single-cell RNA sequencing analysis of the non-myocyte cellular landscape revealed that IRF8 is linked to chronic inflammation in the mouse heart (Skelly et al., 2018). Besides regulation of innate immune responses, IRF8 has been implicated as a tumor suppressor gene in certain cancers (Jiang et al., 2014). A study by Jiang et al. (2014) found that the expression level of IRF8 was down-regulated in the hearts of patients with dilated/hypertrophic cardiomyopathy. Moreover, the cardiac-specific overexpression of IRF8 in mice was found to be protective against aortic banding-induced cardiac hypertrophy. The authors provide mechanistic data to demonstrate that IRF8 interacts with nuclear factor of activated T cells 1 (NFATC1) to prevent nuclear translocation of the latter, and thus inhibits the hypertrophic response. PPARG is a member of the peroxisome proliferator-activated receptor subfamily of nuclear receptors (Mistry and Cresci, 2010). Once activated by a ligand, PPARG binds to its cognate DNA regulatory element as a heterodimer with retinoid X receptors and modulates conformation of the nuclear receptor complex resulting in the association of co-activators, release of co-repressors, and increased transcriptional activation of target genes (Mistry and Cresci, 2010). PPARG has been implicated in the pathology of numerous diseases including obesity, diabetes, atherosclerosis, and cancer (Mistry and Cresci, 2010). PPARG has also been shown to exert cardioprotective properties by suppressing inflammation, oxidative stress and apoptosis, and improving glucose and lipid metabolism (Mirza et al., 2019). Furthermore, when hypercholesterolemic rabbits were pretreated with the PPARG agonist rosiglitazone prior to ischemia/reperfusion or AMI, there was a significant decrease in the number of apoptotic cardiomyocytes and size of myocardial infarct observed (Liu et al., 2004). In a HF rat model, the PPARG agonist, pioglitazone, has also been shown to prevent myocardial fibrosis and HF development through the suppression of the Wnt-β-catenin signaling pathway (Kamimura et al., 2016). NF-κB is a homo- or heterodimeric complex formed by NFKB1/P105 or NFKB2/P52 bound to either REL, P65/RELA, or RELB (Sorriento and Iaccarino, 2019). The heterodimeric P65-NFKB1 complex is the most abundant form of NF-κB. Inappropriate activation of NF-κB initiates the inflammatory cascade and apoptosis-associated genes, which contributes to progressive cardiac dysfunction (Sorriento and Iaccarino, 2019). KLF4 can act both as activator and as repressor that binds the 5′-CACCC-3′ core sequence (Liao et al., 2010). KLF4-deficiency impairs mitochondrial homeostasis and leads to HF development (Liao et al., 2010). Fos can dimerize with proteins of the JUN family, thereby forming the TF complex AP-1. Activation of AP-1 has been shown to be a central component in TGFβ-mediated cardiac fibrosis (Gabriel-Costa, 2018). Collectively, our results revealed that the seven TFs formed a connected regulatory network with genes in the blue module, thus suggesting that the changes in these TF activities may have important roles in the occurrence and progression of post-infarction HF.

Although more than 90% of all mammalian genomes are positively transcribed, less than 2% are subsequently translated into proteins and a large proportion are transcribed as non-protein-coding RNAs (Djebali et al., 2012; Yang et al., 2014). Non-coding RNAs are known to regulate gene expression through diverse mechanisms, such as chromatin remodeling, post-transcriptional regulation, and translational control (Djebali et al., 2012). Among the non-coding RNAs, miRNAs and lncRNAs have been investigated in patients with AMI. A recent study by Li et al. integrated multiple microarray data sets including GSE48060, GSE66360, GSE97320, and GSE19339 to identify miRNAs in patients with AMI and in control subjects (Li et al., 2019b). Four miRNAs, namely, let-7d, let-7b, miR-124-3, and miR-9-1, were predicted to be involved in the pathogenesis of AMI. However, these four miRNAs were not detected in our study. This discrepancy may be attributed to different inclusion criteria for patient selection. Our study found that miR-142-3p regulated a large portion of the genes in the blue module that contributed to the development of HF after AMI. Up-regulating miR-142-3p has been reported to ameliorate myocardial ischemic injury (Su et al., 2019) and attenuate cardiac hypertrophy (Liu et al., 2018a). Recently, Vausort et al. quantified the expression levels of five lncRNAs in patients with AMI using quantitative polymerase chain reaction (Vausort et al., 2014). The results showed that the levels of specific lncRNAs including aHIF, ANRIL, KCNQ10T1, MALAT1, and MIAT in PBMCs were differentially expressed following AMI and may assist in the prediction of cardiac outcomes. However, the selection of these five lncRNAs was subjective. Therefore, an unbiased transcriptional profiling approach would be more informative in identifying many other lncRNAs regulated following AMI, and potentially in discriminating HF development. In our study, through transcriptomics-based screening, we found that the 13 identified lncRNAs exhibited close interactions with genes in the blue module. Studies have revealed that AATBC (Zhao et al., 2015), ADAMTSL4-AS1 (Annunziato et al., 2019), EPB41L4A-AS1 (Liao et al., 2019), MIA-RAB4B (Thean et al., 2017), MIR4697HG (Zhang et al., 2017a), GAS5 (Ni et al., 2019), SNHG12 (Tamang et al., 2019), MSX2P1 (Qiao et al., 2018), and LINC00852 (Liu et al., 2018b) are related to tumorigenesis and tumor progression in multiple cancers, such as bladder, breast, colorectal, and ovarian. Apart from the nine lncRNAs identified in biological experiments, LOC254896 (Singh et al., 2018), LINC01000 (Mitchell et al., 2017), LINC00893 (Li et al., 2019a), and LINC00537 (Li et al., 2017b) were also reported to be significantly differentially expressed between diseased and normal tissues in some transcriptional profiling analyses. Considering their reproducibility across platforms and cohorts, the three transcripts may not be technical artifacts, but rather surrogates of the underlying biology. To date, there are few studies on AMI showing the functional role of the miRNAs and lncRNAs identified in the present WGCNA. Functionally related genes often exhibit similar expression patterns under diverse conditions in DNA microarray experiments (Segal et al., 2003). Thus, analysis of gene co-expression relationships has been considered to be a useful method for exploring the functions of many genes for which information is currently unavailable (Wang et al., 2019). By constructing the miRNA-target regulatory network and lncRNA-mRNA co-expression network, the biological functions of the non-coding RNAs can be inferred from the GO and KEGG pathway enrichment analyses of the known genes in the module. Therefore, the functional annotation of the blue module detected by the WGCNA provided us with a first step toward uncovering functions of the miRNAs and lncRNAs in AMI on a global scale. Additional work will be required to delineate regulatory functions for these non-coding RNAs in the pathological remodeling after AMI.

Our study identified 211 transcripts significantly affected in HF patients, of which six of the most promising, namely, *BCL3*, *HCK*, *PPIF*, *S100A9*, *SERPINA1*, and *TBC1D9B*, were analyzed further. The IκB family member, *BCL3*, was initially identified as a proto-oncogene (Kreisel et al., 2011). Numerous studies have demonstrated that BCL3 not only inhibits the nuclear translocation of the NFκB p50 subunit in the cytoplasm, but also contributes to the regulation of NFκB target genes in the nucleus (Gordon et al., 2011). BCL3 expression has been shown to negatively regulate inflammatory responses through limiting emergency granulopoiesis (Kreisel et al., 2011). Increased expression of NFκB p50 promotes cardiac remodeling and deterioration of cardiac function following AMI (Frantz et al., 2006). Moreover, BCL3 was shown to synergize with PPARG coactivator 1α (PGC-1α) to activate estrogen-related receptor α (ERRα) (Yang et al., 2009). The deactivation of the PGC-1α/ERRα axis are implicated as important mechanisms in the transition from compensated cardiac hypertrophy to HF (Schilling and Kelly, 2011). HCK is a non-receptor tyrosine kinase and plays an important role in the regulation of innate immune responses, phagocytosis, cell survival and proliferation, and cell adhesion and migration (Kovács et al., 2014). HCK has also been described as the key regulator of phagocytosis in macrophages (Tao et al., 2015). When the phagocytosis of apoptotic cardiomyocytes is defective, a persistent proinflammatory state chronically exists in the setting of ischemic injury, thereby predisposing the patients to impaired cardiac function (Tao et al., 2015). PPIF locates in the inner mitochondrial membrane and is involved in regulation of the mitochondrial permeability transition pore (mPTP). The immunosuppressive agent cyclosporin A exhibited significant cardioprotective effects against ischemic injury through inhibiting mPTP formation *via* binding with PPIF (Zhang et al., 2019). S100A9 is a calcium- and zinc-binding protein and is constitutively expressed in neutrophils, dendritic cells, and monocytes. S100A9 deficiency leads to an exacerbated release of cytokines in dendritic cells following stimulation of toll-like receptors (Averill et al., 2011). Additionally, S100A9 has been shown to exert a protective role in preventing exaggerated tissue damage by scavenging oxidants (Gomes et al., 2013). SERPINA1 is the most abundant serine protease inhibitor in human blood and exerts anti-inflammatory and immune-modulatory effects. Genetic SERPINA1 deficiency was associated with increased cardiovascular risk (Curjuric et al., 2018). TBC1D9B, a GTPase-activating protein for Rab family protein, positively regulated autophagic flux by interacting with LC3B (Liao et al., 2018). Taken together, we found that BCL3, HCK, PPIF, S100A9, and SERPINA1 may have important roles in the development of HF through regulating local and systemic inflammation. Furthermore, the ROC analysis has indicated that the six identified hub genes are likely to be good biomarkers with high sensitivity and specificity for HF prognosis in patients with AMI. Further studies and experimental verification are needed to determine clinical usefulness of the six hub genes as a non-invasive test for prognosis of HF development.

This study is significant in that it identifies several key genes involved in HF development in post-AMI patients via comprehensive bioinformatics methods. To our knowledge, this was the first study to identify key lncRNAs associated with the development of post-infarction HF through the use of probe re-annotation and the WGCNA algorithm. These findings may serve to clarify the pathophysiology of cardiac remodeling and to identify blood biomarkers in patients at high risk for the development of HF following AMI. However, mRNA levels and protein expression of the identified genes, including 7 TFs, 26 miRNAs, 13 lncRNAs, and 6 hub genes were not confirmed via further experiments. Additionally, the probe re-annotation in microarrays is not capable of identifying all lncRNAs, and thus a deep RNA sequencing experiment that encompasses cardiac coding (mRNA) and non-coding (miRNA and lncRNA) transcriptomes will provide a more detailed understanding of the myocardial transcriptome landscape in HF after AMI.

## Conclusions

Our study used the WGCNA to construct a gene co-expression network, and to identify and validate the key modules and hub genes associated with the development of HF after AMI. Six hub genes, including *BCL3*, *HCK*, *PPIF*, *S100A9*, *SERPINA1*, and *TBC1D9B* that differentiated on admission after myocardial infarction the HF patients from the non-HF ones, can serve as early prognostic biomarkers of post-AMI patients. Moreover, seven TFs (SPI1, ZBTB7A, IRF8, PPARG, P65, KLF4, and Fos), as well as miR-142-3p, and LINC00537 were predicted to regulate the key genes that contributed to the pathophysiological consequences of AMI. Although our investigation is of a preliminary nature, this study provides new insights into the pathogenesis of HF following AMI and may, therefore, have significant implications for potential therapeutic targets of AMI.

## Data Availability Statement

All datasets for this study are included in the article.

## Author Contributions

XN and ZZ conceived and designed the study. XN, JZ, LZ, YH, SP, AC, and MB performed the study. XN, LZ, YH, SP, and AC analyzed the data. XN and ZZ wrote the manuscript. JZ, LZ, YH, SP, AC, and MB critically revised intellectual content of the manuscript.

## Funding

The authors acknowledge financial support from Research Project of Gansu Provincial Administration of Traditional Chinese Medicine (GZK-2018-48). Undergraduate Training Program for Innovation and Entrepreneurship of Lanzhou University (20190060188), and Excellent Plan for Student Scientific Research Innovation Cultivation Project (20190060188).

## Conflict of Interest

The authors declare that the research was conducted in the absence of any commercial or financial relationships that could be construed as a potential conflict of interest.

## Supplementary Material

Supplementary Figure S1 Clustering of samples and determination of soft-thresholding power in GSE59867. **(A)** Clustering dendrogram of 65 samples in the dataset GSE59867. Red, Yes; Green, No. **(B)** Analysis of the scale-free fit index for various soft-thresholding powers (β). **(C)** Analysis of the mean connectivity for various soft-thresholding powers.

Supplementary Figure S2 Identification of distinct modules for co-expressed genes in AMI using WGCNA in GSE59867.

Supplementary Figure S3 Clustering of samples and determination of soft-thresholding power in GSE42955. **(A)** Clustering dendrogram of 16 samples in the dataset GSE42955. Red, Yes; Green, No. **(B)** Analysis of the scale-free fit index for various soft-thresholding powers (β). **(C)** Analysis of the mean connectivity for various soft-thresholding powers.

Supplementary Figure S4 Identification of distinct modules for co-expressed genes in AMI using WGCNA in GSE42955.
