# Supplementary figures and images for: Weighted Gene Co-Expression Network Analysis Identifies Critical Genes in the Development of Heart Failure After Acute Myocardial Infarction
Source: Front Genet. 2019 Nov 26;10:1214. doi: 10.3389/fgene.2019.01214 (PMC6889910; doi:10.3389/fgene.2019.01214)

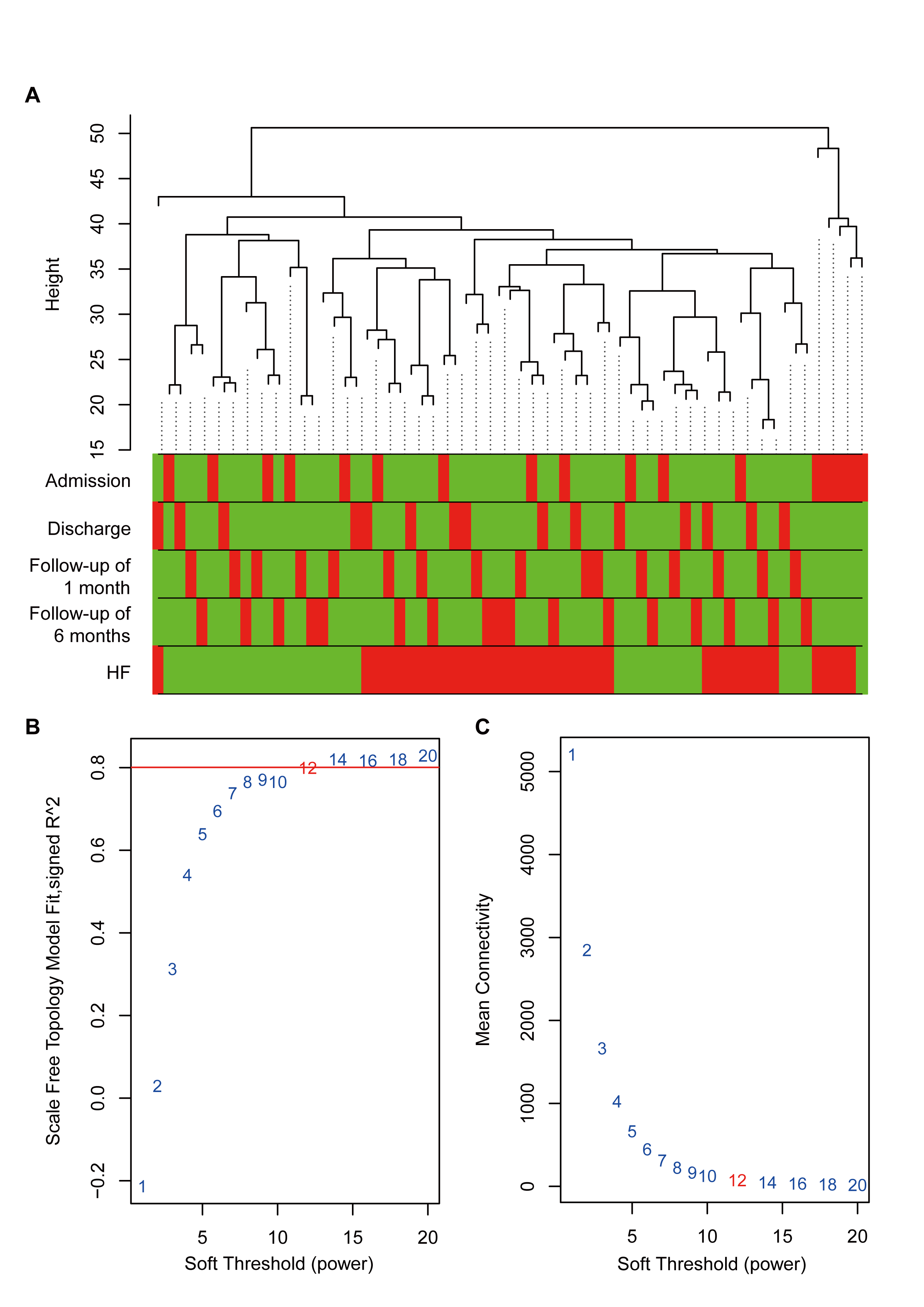

Supplement: Supplementary Figure S1 — Clustering of samples and determination of soft-thresholding power in GSE59867. (A) Clustering dendrogram of 65 samples in the dataset GSE59867. Red, Yes; Green, No. (B) Analysis of the scale-free fit index for various soft-thresholding powers (β). (C) Analysis of the mean connectivity for various soft-thresholding powers. [file Image_1.tif]

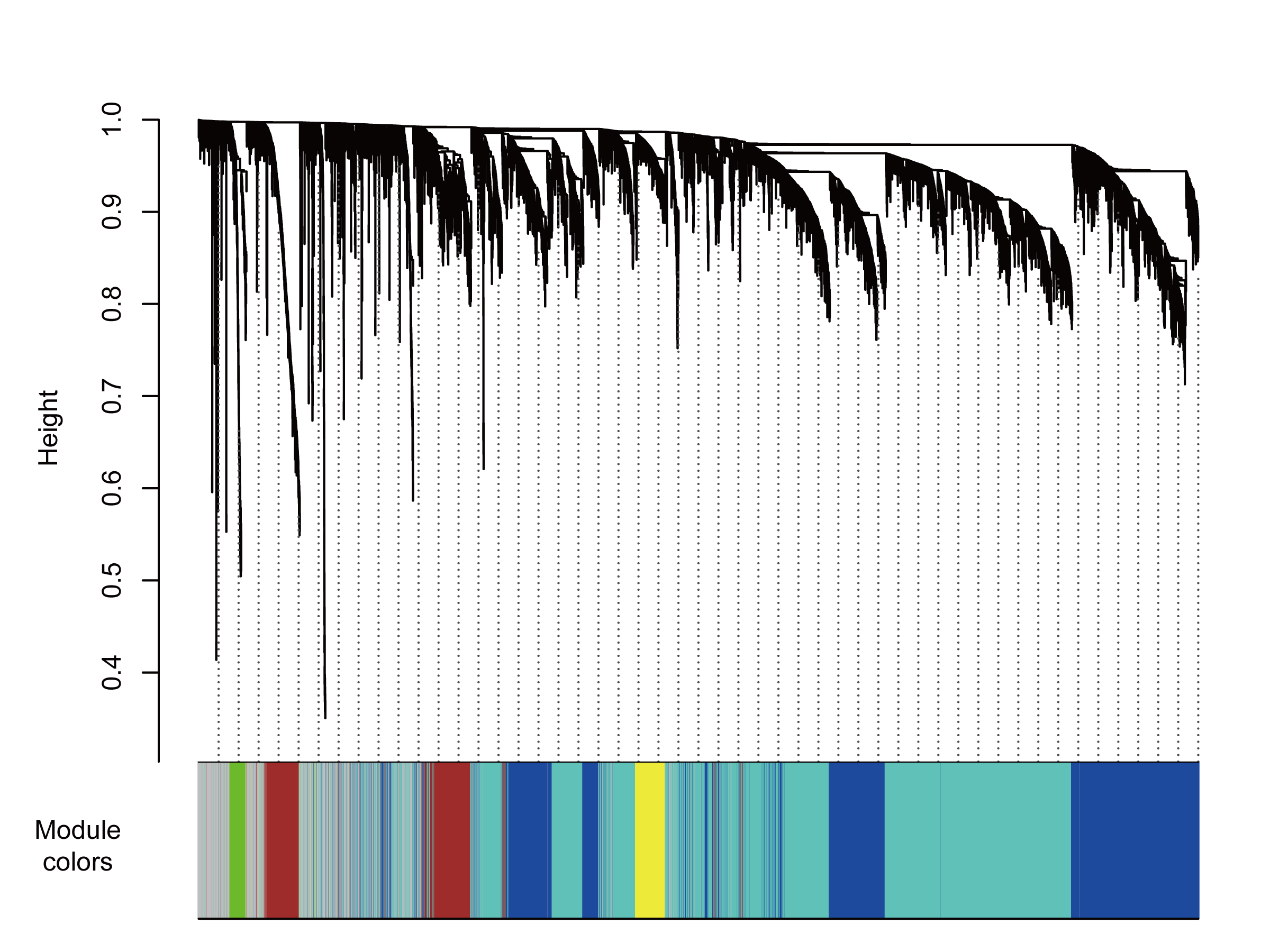

Supplement: Supplementary Figure S2 — Identification of distinct modules for co-expressed genes in AMI using WGCNA in GSE59867. [file Image_2.tif]

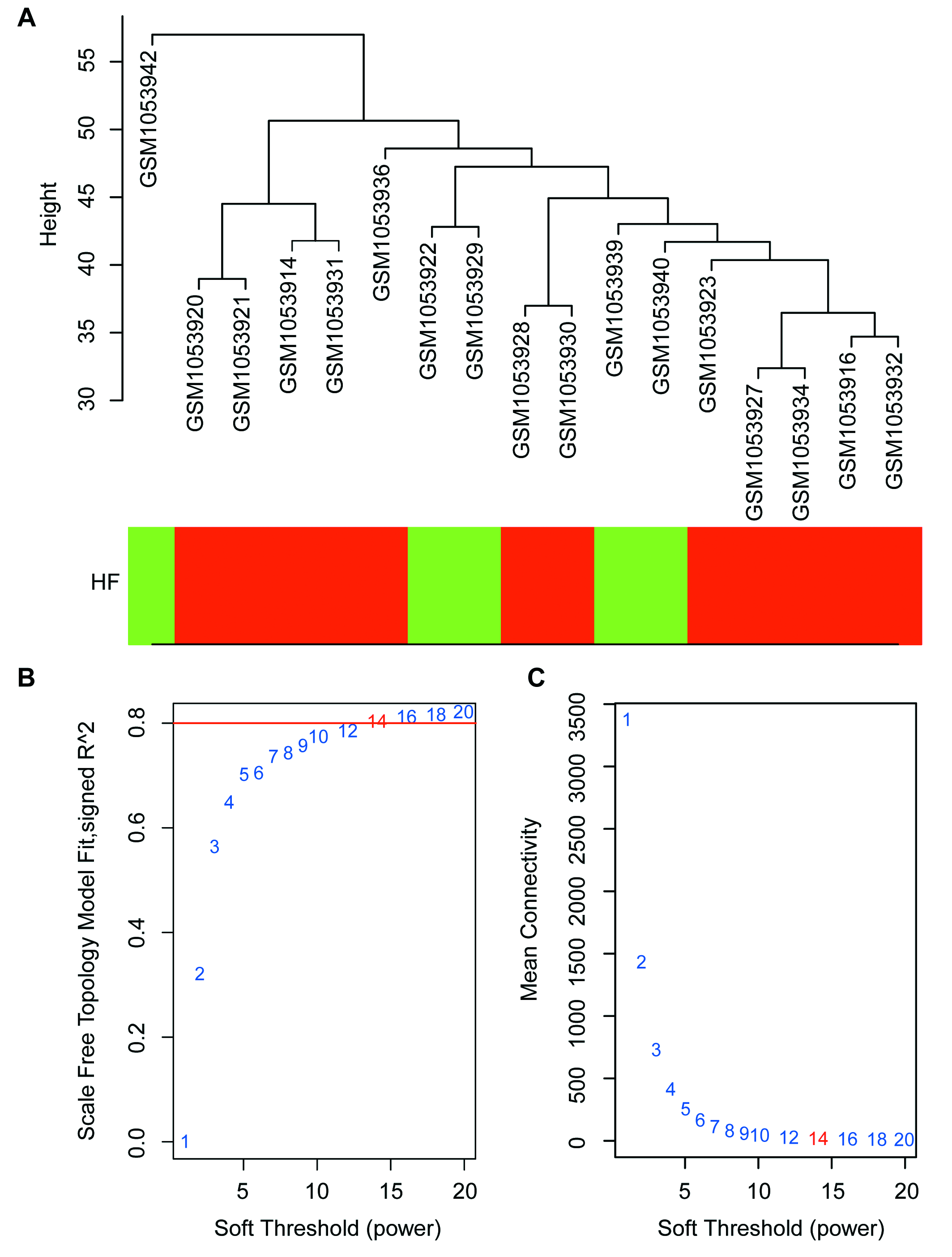

Supplement: Supplementary Figure S3 — Clustering of samples and determination of soft-thresholding power in GSE42955. (A) Clustering dendrogram of 16 samples in the dataset GSE42955. Red, Yes; Green, No. (B) Analysis of the scale-free fit index for various soft-thresholding powers (β). (C) Analysis of the mean connectivity for various soft-thresholding powers. [file Image_3.tif]

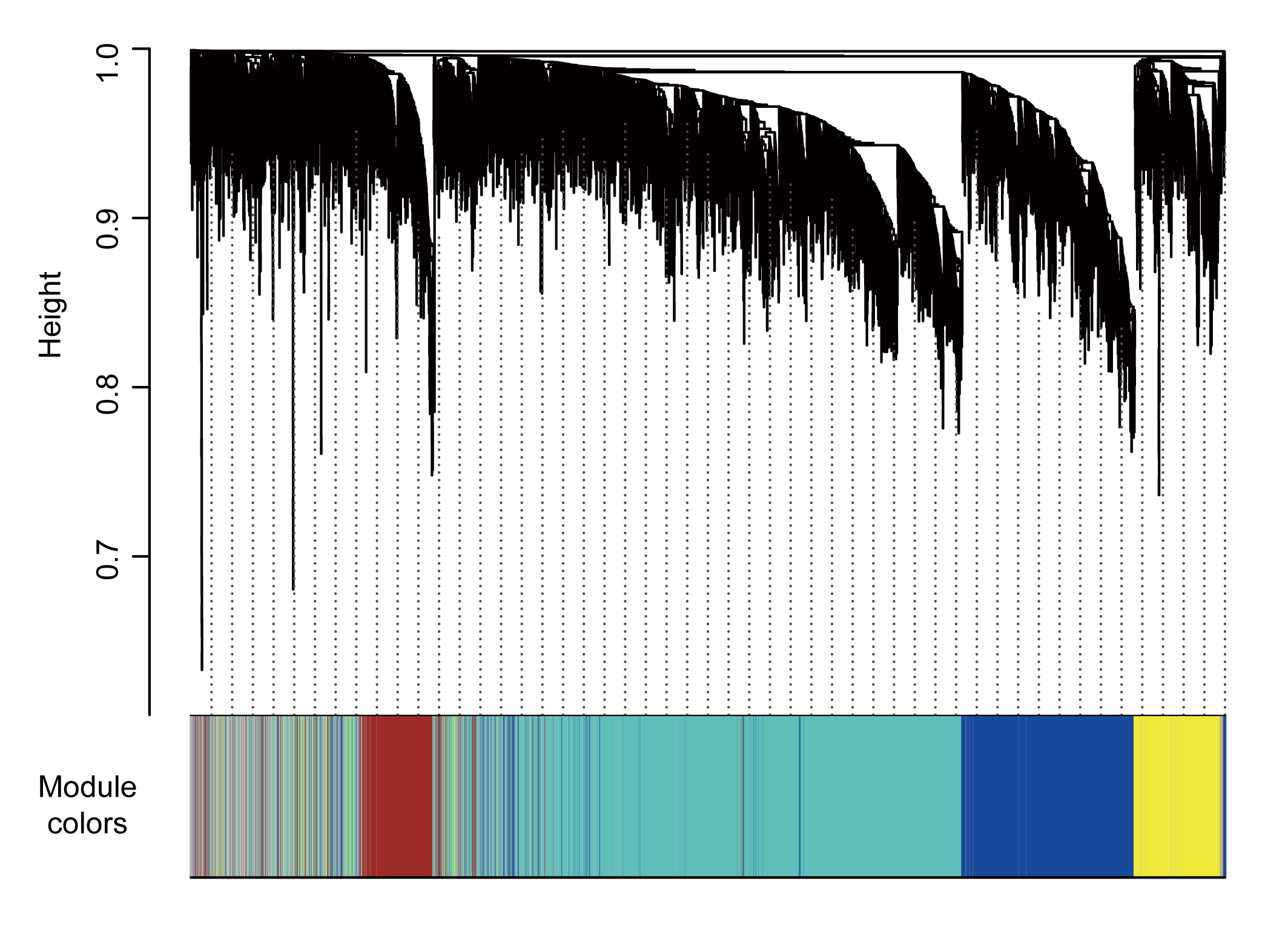

Supplement: Supplementary Figure S4 — Identification of distinct modules for co-expressed genes in AMI using WGCNA in GSE42955. [file Image_4.tif]
