# Supplementary material for: Weighted Gene Co-Expression Network Analysis Identifies Critical Genes in the Development of Heart Failure After Acute Myocardial Infarction
Source: Front Genet. 2019 Nov 26;10:1214. doi: 10.3389/fgene.2019.01214 (PMC6889910; doi:10.3389/fgene.2019.01214)
Supplement: Supplementary file 5 [file DataSheet_1.pdf]

```
library(WGCNA)
library(flashClust)
dis= read.csv("WGCNA.csv")
dim(dis)
names(dis)
rownames(dis) <- dis$X
datExpr0= as.data.frame(t(dis[, -c(1)]))
names(datExpr0)= dis$X
rownames(datExpr0)=names(dis)[-c(1)]
dim(datExpr0)
gsg=goodSamplesGenes(datExpr0, verbose = 3)
gsg$allOK
traitData= read.csv("traits.csv")
dim(traitData)
head(traitData)
names(traitData)
rownames(traitData) <- traitData$Sample
traitData$Sample <- NULL
datTraits= traitData
A=adjacency(t(datExpr0),type="unsigned")
k=as.numeric(apply(A,2,sum))-1
Z.k=scale(k)
thresholdZ.k=-2.0
outlierColor=ifelse(Z.k<thresholdZ.k,"red","black")
sampleTree = flashClust(as.dist(1-A), method = "average")
traitColors=data.frame(numbers2colors(datTraits,signed=FALSE))
dimnames(traitColors)[[2]]=paste(names(datTraits))
datColors=data.frame(outlier=outlierColor,traitColors)
quartz()
plotDendroAndColors(sampleTree,groupLabels=names(datColors),
                    colors=datColors,main="Sample dendrogram and trait heatmap")
remove.samples= Z.k<thresholdZ.k | is.na(Z.k)
datExpr0=datExpr0[!remove.samples,]
datTraits=datTraits[!remove.samples,]
A=adjacency(t(datExpr0),type="distance")
k=as.numeric(apply(A,2,sum))-1
Z.k=scale(k)
save(datExpr0, datTraits, file="SamplesAndTraits.RData")
options(stringsAsFactors = FALSE)
lnames= load(file="SamplesAndTraits.RData")
lnames
dim(datExpr0)
dim(datTraits)
powers= c(seq(1,10,by=0.5), seq(from =12, to=40, by=2))
```

```
sft = pickSoftThreshold(datExpr0, powerVector=powers, verbose =5,networkType="unsigned")
quartz()
par(mfrow= c(1,2))
cex1=0.9
plot(sft$fitIndices[,1], -sign(sft$fitIndices[,3])*sft$fitIndices[,2], xlab= "Soft Threshold (power)", ylab="Scale Free Topology Model Fit, signed", type= "n", main= paste("Scale independence"))
text(sft$fitIndices[,1], -sign(sft$fitIndices[,3])*sft$fitIndices[,2], labels=powers, cex=cex1, col="red")
abline(h=0.80, col="red")
plot(sft$fitIndices[,1], sft$fitIndices[,5], xlab= "Soft Threshold (power)", ylab="Mean Connectivity", type="n", main = paste("Mean connectivity"))
text(sft$fitIndices[,1], sft$fitIndices[,5], labels=powers, cex=cex1, col="red")
adjacency=adjacency(datExpr0, power=softPower, type="unsigned")
TOM= TOMsimilarity(adjacency, TOMType="unsigned")
dissTOM= 1-TOM
geneTree= flashClust(as.dist(dissTOM), method="average")
quartz()
plot(geneTree, xlab="", sub="", main= "Gene Clustering on TOM-based dissimilarity", labels= FALSE, hang=0.04)
dynamicMods= cutreeDynamic(dendro= geneTree, distM= dissTOM, deepSplit=2, pamRespectsDendro= FALSE, minClusterSize= minModuleSize)
table(dynamicMods)
dynamicColors= labels2colors(dynamicMods)
quartz()
plotDendroAndColors(geneTree, dynamicColors, "Dynamic Tree Cut", dendroLabels= FALSE, hang=0.03, addGuide= TRUE, guideHang= 0.05, main= "Gene dendrogram and module colors")
MEList= moduleEigengenes(datExpr0, colors= dynamicColors)
MEs= MEList$eigengenes
MEDiss= 1-cor(MEs)
METree= flashClust(as.dist(MEDiss), method= "average")
quartz()
plot(METree, main= "Clustering of module eigengenes", xlab= "", sub= "")
abline(h=MEDissThres, col="red")
merge= mergeCloseModules(datExpr0, dynamicColors, cutHeight= MEDissThres, verbose =3)
mergedColors= merge$colors
mergedMEs= merge$newMEs
quartz()
plotDendroAndColors(geneTree, cbind(dynamicColors, mergedColors), c("Dynamic Tree Cut", "Merged dynamic"), dendroLabels= FALSE, hang=0.03, addGuide= TRUE, guideHang=0.05)
moduleColors= mergedColors
colorOrder= c("grey", standardColors(50))
moduleLabels= match(moduleColors, colorOrder)-1
MEs=mergedMEs
save(MEs, moduleLabels, moduleColors, geneTree, file= "SamplesAndColors.RData")
```

```
datt=datExpr0
nGenes = ncol(datt);
nSamples = nrow(datt);
MEs0 = moduleEigengenes(datt, moduleColors)$eigengenes
MEs = orderMEs(MEs0)
moduleGeneCor=cor(MEs,datt)
moduleGenePvalue = corPvalueStudent(moduleGeneCor, nSamples);
moduleTraitCor = cor(MEs, datTraits, use = "p");
moduleTraitPvalue = corPvalueStudent(moduleTraitCor, nSamples);
quartz()
textMatrix = paste(signif(moduleTraitCor, 2), "\n(", signif(moduleTraitPvalue, 1), ")", sep = "");
dim(textMatrix) = dim(moduleTraitCor)
par(mar = c(6, 8.5, 3, 3));
labeledHeatmap(Matrix = moduleTraitCor, xLabels = names(datTraits), yLabels = names(MEs),
ySymbols = names(MEs), colorLabels = FALSE, colors = blueWhiteRed(50), textMatrix =
textMatrix, setStdMargins = FALSE, cex.text = 0.7, zlim = c(-1,1), main = paste("Module-trait
relationships"))
whichTrait="HF"
quartz()
nGenes = ncol(datt);
nSamples = nrow(datt);
selTrait = as.data.frame(datTraits[,whichTrait]);
names(selTrait) = whichTrait
modNames = substring(names(MEs), 3)
geneModuleMembership = as.data.frame(signedKME(datt, MEs));
MMPvalue = as.data.frame(corPvalueStudent(as.matrix(geneModuleMembership), nSamples));
names(geneModuleMembership) = paste("MM", modNames, sep="");
names(MMPvalue) = paste("p.MM", modNames, sep="");
geneTraitSignificance = as.data.frame(cor(datt, selTrait, use = "p"));
GSPvalue = as.data.frame(corPvalueStudent(as.matrix(geneTraitSignificance), nSamples));
names(geneTraitSignificance) = paste("GS.", names(selTrait), sep="");
names(GSPvalue) = paste("p.GS.", names(selTrait), sep="");
par(mfrow=c(2,3))
counter=0
for(module in modNames[1:length(modNames)]){
  counter=counter+1
  if (counter>6) {
    quartz()
    par(mfrow=c(2,3))
    counter=1
  }
  column = match(module, modNames);
  moduleGenes = moduleColors==module;
  verboseScatterplot(abs(geneModuleMembership[moduleGenes, column]),
```

```
                              abs(geneTraitSignificance[moduleGenes, 1]),
                              xlab = paste(module,"module membership"),
                              ylab = paste("GS for", whichTrait),
                              col = module,mgp=c(2.3,1,0))
}
which.module="blue"
datME=MEs
datExpr=datt
quartz()
ME=datME[, paste("ME",which.module, sep="")]
par(mfrow=c(2,1), mar=c(0.3, 5.5, 3, 2))
plotMat(t(scale(datExpr[,moduleColors==which.module ]) ),
          nrgcols=30,rlabels=F,rcols=which.module,
          main=which.module, cex.main=2)
par(mar=c(5, 4.2, 0, 0.7))
barplot(ME, col=which.module, main="", names.arg=c(row.names(datt)), cex.names=0.5,
cex.main=2, ylab="eigengene expression",xlab="sample")
```
